# Dutch tweets before, during, and after a black lives matter demonstration in Amsterdam: Expert annotated data, protocol, and labelling tool

**DOI:** 10.1016/j.dib.2024.110527

**Published:** 2024-05-16

**Authors:** Laurens H.F. Müter, Christof van Nimwegen, Remco C. Veltkamp

**Affiliations:** a Netherlands Police, Van Deventerlaan 50, 3528 AE, Utrecht, the Netherlands; b Utrecht University, Heidelberglaan 8, 3584 CS, Utrecht, the Netherlands

**Keywords:** Social media analysis, Protest, COVID-19, Natural language processing, Manual labelling

## Abstract

The Netherlands police are looking for measures to examine sentiment on social media related to protest demonstrations. While models exist to detect more subtle expressions of sentiment within tweets, models trained in the Dutch language are scarce. Being able to predict sentiment development during protests is relevant for parties like the Dutch government and the police to get more insight to when and where potential law enforcement is needed for public order and safety. Therefore, to analyse sentiment before, during, and after protest demonstrations, data was collected with tweets related to a Black Lives Matter protest that took place in Amsterdam during the COVID-19 pandemic. All tweets have been manually labelled by a dedicated open-source intelligence (OSINT) team within the Netherlands police following an established protocol. Both the data and the protocol are available, and interesting for researchers in natural language processing, topic detection, sentiment analysis, and protests analysis. The developed labelling tool for the labelling process is publicly available.

Specifications Table

**Table:** 

Subject	Data Science, Policy and Law.
Specific subject area	Text mining and manual labelling of tweets related to a Black Lives Matter protest in the Netherlands.
Type of data	Text (CSV file). *Raw, labelled and/or annotated*
Data collection	The tweets were collected within three days after the protest (June 1, 2020). The tweets were collected up to two weeks before the demonstration. In the months after collection, the tweets are labelled by a specially trained team of labellers at the Netherlands police, using a labelling protocol. This protocol is based on interviews with related teams within the Netherlands police. The data is queried with the Twitter API using the keywords “protest”, “black lives matter” and “amsterdam”. Retweets are ignored, and language is set to select Dutch tweets, where full-text tweets and meta-data are included. However, to ensure consistency and readability, all tweet texts are translated into English within this article. Entities such as hashtags and username mentions are included, whereas the latter are hashed to protect the user's privacy. The creation date and a URL to the original content are included. Metadata, such as the number of retweets, friends count, followers, status, and verified versus non-verified users are included.
Data source location	The data was collected from the Netherlands, using the credentials from a free Twitter API account. The data is stored on a Dutch server at Utrecht University for at least 10 years.
Data accessibility	Repository name: data brief am Data identification number: doi: 10.24416/UU01-W9RS5V Direct URL to data: https://public.yoda.uu.nl/science/UU01/W9RS5V.html Instructions for accessing these data: Data are accessible to researchers upon reasonable request for data sharing. To access the data, please send an email to the main author (l.h.f.muter@uu.nl) or the Department of Information and Computing Sciences at Utrecht University (ics.ict@uu.nl).
Related research article	M. Loerakker, L. Müter, M. Schraagen, Finetuning language models on Dutch protest event tweets. In *Proceedings of the 7th Workshop on Challenges and Applications of Automated Extraction of Socio-political Events from Text, CASE 2024 (2024)* 6–23.

## 1 Value of the Data

• The data is labelled by experts in a dedicated open source intelligence (OSINT) team within the Netherlands police following an established protocol.
• The data is of value for researchers in the fields of natural language processing, topic detection, sentiment analysis, and protests analysis.
• Research focusing on tweets or protest demonstrations can utilise these data sets. Also, the protocols and tooling used to label the data can be useful for future labelling.
• The data set facilitates our research on how events evolve during protest demonstrations, which gives the government and the police insight into when and where potential law enforcement is needed for public order and safety.
• Dutch language models are scarce, since little data is available for training and testing. This data set can contribute by adding valuable data related to a real-world protest.

## 2 Background

### 2.1 Large-scale event planning

To estimate the social impact of protest demonstrations, the OSINT team tries to gain insights into topics related to the social movement that underlies upcoming protest demonstrations. For the analysis of these topics, multiple open and closed sources are used. Thus, Twitter should be considered as an isolated source that is solely used to make predictions about the cause of an upcoming protest.

In most cases, the OSINT team responds to information requests from other departments within the police, where information requests related to large-scale events are most common. Since most of the OSINT work is demand-driven, there is often prior information available, where OSINT can use this initial information as a starting point to query available information sources.

The primary focus of the OSINT team often is obtaining a global view of discussed topics concerning an upcoming protest demonstration. For these topics, the OSINT team is also interested in how people will act during the demonstration. For example, when people are expected to behave aggressively, additional measures can be considered to protect public safety. In other cases, a large crowd might be very critical of a public spokesman, and additional measures can be taken to safeguard this person.

To gain insights into aggressive behaviour during a protest, we suggest the use of BERT-based models to detect expressions of discontent [9]. Although research is done on sentiment analysis in tweets, there is still little literature on Dutch tweets within the context of protest demonstrations. Thus, this data set is created to shed more light on the construction of sentiment models related to Dutch protest using tweets. After carefully labelling a subset of the data, multiple BERT models (Bernice, Bertje, TwHIN-BERT-base, TwHIN-BERT-large, and mBERT) are selected and fine-tuned on 80 % of the labelled tweets. After testing the models on the remaining 20 % we found that Bernice and TwHIN-BERT-base scored the most promising on sentiment prediction, with F1 scores ranging from 0.632 to 0.663 for Bernice and 0.635 to 0.656 for TwHIN-BERT-base [9].

### 2.2 Case: black lives matter demonstration

Various media [[1], [2], [3], [4]] and a research report commissioned by the government of Amsterdam [5] have been consulted to make a detailed description of the activities leading toward the demonstration and the different events during the demonstration.

The Black Lives Matter demonstration was scheduled on June 1, 2020, at Dam Square in Amsterdam. In the days before this demonstration, involved organizations were busy planning and making arrangements related to the demonstration, which can be seen in a Facebook event by Black Queer & Trans Resistance NL ‘Solidarity protest against anti-black violence in the US and EU’ [6]. Moreover, the police were aware of the upcoming BLM demonstrations days before (e.g. [5]). Worldwide collective anger, triggered by a violent police crackdown in the U.S. city of Minneapolis in which the black citizen George Floyd lost his life, provoked a chain reaction that led to demonstrations in large cities around the world. The demonstrations in the United States also resulted in discussions on multiple WhatsApp groups within anti-racism organizations such as Black Lives Matter and Kick Out Zwarte Piet, where ideas were suggested to commemorate George Floyd [1].

### 2.3 Saturday, May 30, 2020

The first sketches of the demonstration began to take shape in multiple WhatsApp groups and on social media such as Facebook, where discussion regarding police violence and discrimination fuelled the need to start a demonstration [3].

### 2.4 Sunday, May 31, 2020

Some prominent figures in the anti-racism community and the Kick Out Zwarte Piet collective decided to organize a demonstration on Monday afternoon in Amsterdam. A Facebook event was started where hundreds of registrations came in [3]. According to Boin et al., [5], the Black Lives Matter organization “Nederland Wordt Beter” notified the municipality of Amsterdam at 16:52 about an upcoming demonstration against police violence and discrimination. Officials of the Public Order and Safety Department of the municipality of Amsterdam were appointed to estimate the number of people joining the demonstration. The organizers of the demonstration were contacted to acquire their expectations and whether they could guarantee a minimum distance of one and a half meters between the protesters. At that moment, the organizers expected hundred fifty to three hundred people and promised to draw markings on the pavement to ensure that protesters would keep their distance. At that time, however, there was already a Facebook initiative from an individual related to this demonstration where more than a thousand people had registered. Since this event was not linked to the upcoming demonstration, the number of attendees was underestimated by the organizers. The police officials also based their predictions on Black Lives Matter protests in other cities such as London (hundreds of protesters) and Berlin (less than fifteen hundred protesters). Additional safety measures were taken, such as plain clothes police officers, but those measures were mainly focused on preventing violence from counterdemonstrations for instance right-wing groups. The police also scanned open sources such as newspaper articles and social media to improve their estimates [3].

### 2.5 Monday, June 1, 2020

At noon, a briefing with the mayor of Amsterdam and the unit commander of the Amsterdam police took place. At that time, the police were expecting more than 600 protesters, so they considered notifying the chief public prosecutor for an official consultation. However, an official consultation did not take place because there was good contact with the organizers of the demonstration, and they appeared to take their responsibility regarding the safety of the protesters seriously [5]. Moreover, the mayor of Amsterdam refrained from imposing additional restrictions on the demonstration [3]. Initially, the demonstration would have taken place at a large greensward near Museumplein. However, the organizers still expected a relatively small group of protesters, so the smaller Dam Square in the centre of Amsterdam was selected as the venue. The thriving force behind the Black Lives Matter demonstration [2] arrived around 16:30 at the Dam Square where a small group of people was busy drawing markings on the pavement for the 500 expected protesters. There were also aisles and signs to remind protesters to comply with the coronavirus measures. In the afternoon, announcements of the manifestation were attracting thousands of people on Facebook, and the police estimated a maximum of a thousand protesters at 16:30. Once the speakers’ program started at 17:00, more people found their way to Dam Square. In a short amount of time, more than 5000 people were gathered at the demonstration. The police blocked public transport and car traffic in an attempt to stop the influx of people. The police did not take any action to end the demonstration, because the coronavirus measure to prohibit gatherings was discontinued at noon that day [5]. Moreover, any action to end the demonstration could lead to violence, and the right to demonstrate is a fundamental right by Dutch law [7].

## 3 Data Description

The labels used to classify the tweets are mainly based on the needs of the OSINT unit of the Netherlands police as presented in Table 1. Multiple ‘label types’ are defined, to add a semi-hierarchical structure within the labels (see also Table 2);

1. Relevance: the contents of the tweet might be relevant to law enforcement.
2. Event Related: referring to a legal activity related to the main topic of the data set.
3. Incident Related: where an act of violation of the Dutch law or (temporary) regulations is described, such as a violation of COVID-related rules or violence.
4. Priority: indicates whether the tweet is relevant for crisis management. This label indicates a superlative form of the relevance label.
5. Incident Type: if the Incident Related label is applicable on a tweet, then this label can specify the type of the incident. This label encompasses three types: Corona, Violence, and Riot.
6. Expression of Discomfort: to indicate whether a tweet contains expressions of distress, annoyance, uneasiness, or anger.
7. No label: to indicate when none of the above labels applies.

**Table 1:** Descriptions of the labels and label types used within this set.

Relevance	Boolean	Indicates if a tweet contains relevant information for OSINT analysts (1) or not (0)
High_Priority	Boolean	Indicates if the content of a tweet requires direct action (1) or not (0).
Incident_Related	Boolean	Tweet text is related to an incident (1) or (0).
Violence	Boolean	Tweet contains text related to violent behaviour (1) or not (0)
Discomfort	Boolean	Tweet contains an expression of discomfort (1) or not (0)
Corona	Boolean	Tweet contains a reference to SARS COVID-19 (1) or not (0)
Riot	Boolean	Tweet contains textual information about rioting behaviour (1) or not (0)
Event_Related	Boolean	Tweet refers to an event or happening during the protest (1) or not (0)
Tweet_ID	Numeric	Unique identifier provided by Twitter
Last_Label_Update_At	Date Time	Date time of the last update in the label is made
Number_Of_Updates	Numeric	Number of times the tweet has been updated by labeller(s)
Label_User_Hash	String	Hashed value of the labeller that labelled the tweet
Lang	String	Language code of the tweet, provided by Twitter
Possibly_Sensitive	Boolean	Tweet contains sensitive information (1) or not (0), as provided by Twitter
No_Labels	Boolean	When none of the above labels is applied, the No_label is set to (1) else (0)

**Table 2:** Descriptions of the categories of used labels within this set.

Category	Labels	Description
Relevance	*Other_Relevance_Type*	Information contained in this tweet is relevant for law-keepers
Incident_Related	*Corona, Other_Incident, Riot, Violence*	Information contained in this tweet refers to an illegal activity during the protest
Behavioural_Relevance	*Discomfort*	Tweet contains an expression of discomfort that is directly or indirectly related to the protest
Event_Related	*Event_Related*	description of a non-illegal activity
High_Priority	*High_Priority*	Tweet might result in direct action from law enforcement officers

For the Incident Type label, it was allowed to assign more than one of its subcategories to a tweet. For the other labels, this was not allowed due to their binary nature: they were either applicable or not.

### 3.1 Unlabelled data

The data is subdivided into multiple separate data sets based on information extracted from the tweet text. Each subsection represents a data set which is included as a CSV text formatted file that contains the given columns and rows as presented in each subsection.

### 3.2 Tweets generic

This set contains generic information about each tweet. This set includes references to original messages and users, represented by the columns: *Tweet_Id, User_Id*, and *Org_Tweet_User*. Date Time is also included in the column *Created_At_TZ* and *Org_Tweet_Created_At*. Other meta-data retweet counts, and favourite counts are also included (*Favourite_Count* and *Retweet_Count*, respectively). This set contains 84,901 rows.

### 3.3 Tweets created At

The date and time are in JSON format to indicate when the tweet was sent. This information is provided by Twitter. The columns include *Tweet_Id* and *Created_At*. This set contains 84,901 unique tweets.

### 3.4 Tweet users

This set contains the hashed username of the account that has sent the tweet. The hashing was done with the Python hash library. Since the seed is consistent, each user's hash is unique. Thus, when a user has sent multiple tweets, the hash value remains consistent for both tweets. The columns include *Tweet_Id* and *User_Id*. This set contains 27,189 unique users.

### 3.5 Retweet original created At

This set solely contains information on the tweets that are retweeted. As such, the creation date and time of the original tweet–that is being retweeted– are provided. The following columns are included: *Original_Tweet_Id* representing the ID from the retweeted tweet, and *Org_Tweet_Created_At* representing the date and time when the original tweet was created. This set contains 55,849 rows.

### 3.6 Retweet original users

The Twitter users who posted tweets that were retweeted are presented within this set. The set represents a hashed value of the user that has sent the original tweet(s) retweeted by others. The columns include *Tweet_Id* and *Original_User_Id*. This set contains 55,849 rows and 3486 unique users.

### 3.7 Hashtags

This set contains the hashtags extracted from the tweet text using regular expressions3. The columns include *Tweet_ID* and *Hashtag*, where *Tweet_ID* might occur multiple times when the tweet contains more than one hashtag. tweets that do not include any hashtags are not included in this data set. This set contains 32,327 rows and 1,788 unique hashtags.

### 3.8 Hashtag connections

This set contains the hashtags extracted from the tweet text using regular expressions. Given that a tweet can contain multiple hashtags, a tweet ID can have multiple occurrences within the data set. The columns include *Tweet_Id* and *Hashtags*. This set contains 8,538 rows.

### 3.9 Mentions

The data includes two data sets that contain references to Twitter users (known as mentions); mention connections and user to mentions. The mentions extracted from the tweet text are represented as text. Comparable to hashtags, the mentions are extracted using regular expressions (The regular expressions used to extract certain features from tweet texts are included in the pre-processing notebook which is included in the data repository).

In the mention connections data set, each row represents two co-occurring mentions. Hence, a single tweet can span multiple rows (for all combinations of mentions). The columns include Tweet Id and Mention Id, where the mention ID corresponds to the user ID when the username and mention name are equal. This set contains 94,797 rows and 11,179 unique mentions. In this data set, the Twitter user who posted the tweet serves as a source, and each mentioned user serves as a target (sometimes multiple mentioned users). The user to mentions data set contains the relation between a Twitter user who posted the tweet and a user mentioned within the posted tweet. Note that a single tweet could be represented in multiple rows since each row contains one user-mention pair. The included columns contain *Tweet_ID, User_ID*, and *Mention_Id*. This set contains 55,849 rows.

### 3.10 Emojis

This set contains textual representations of emojis extracted from the tweet text using the Python library Emoji. Since a tweet can contain multiple emojis, the emojis are presented as a JSON list object. This set consist of 4,296 rows and contains the columns *Tweet_ID* and *Emoji*.

### 3.11 Emojis connections

The extracted emojis are listed when they occur in the same tweet. A tweet is excluded when it only contains a single emoji or no emojis at all. The columns include *Tweet_ID* and *Emojis* and the data set contains 892 rows.

### 3.12 Labelled data sets

The second group of filtered data sets contains information extracted from labelled tweets. These sets include textual constructs from the labelled tweets such as hashtags and emojis.

### 3.13 Labels

This set contains the labels per tweet, including the hashed username of the labeller that has assigned the final labelling to the tweet and whether the tweet is discussed during a weekly label meeting. The columns include *Tweet_ID, Label 0*… *Label N, Labeller* and *Is_Discussed*.

### 3.14 Annotations

This set contains the annotations for each applicable tweet and the hashed username of the labeller that was assigned, the annotated tweet, and the labels. The columns are *Tweet_ID, Annotation, Label*, and *labeller*.

### 3.15 Label update history

This set contains the updates on the labels per tweet, where every interaction that changes the labels is considered an update. An update can be executed by a single labeller, e.g. when the labeller presses the back button to correct a label, or by a different labeller, like when pre-labelled tweets are presented. The columns include *Tweet_ID, User_Hashed, Labels*, and *Labled_At*, where *Labelled_At* is a JSON formatted Date Time indication of when the provided label is assigned. The Labels column represents the resulting labels (as a list) after the users’ action that has changed the labels.

## 4 Experimental Design, Materials and Methods

This section covers the main steps in the data collection, pre-processing, and labelling process. Additional materials and methods, including a dedicated tool to support the labelling process, are discussed.

### 4.1 Data collection

The data used in this study comprises Dutch tweets related to protests that occurred between 2020 and 2022. The data retrieval process took place shortly after each incident, utilizing the Twitter API. Due to the API's limitation on historic tweets, the available dataset spans two to three days before and after the protest incidents. On the protest days, the majority of tweets were extracted, and a filter was implemented to specifically select Dutch tweets on protests. Retweets were excluded from the dataset. The Twitter API's free version was employed, resulting in the availability of only about 2 % of all tweets.

Initially, the dataset consisted of 84,901 tweets (see Table 3), including 55,849 retweets. In total, there are 6,155 tweets labelled, representing 21.19 % of the non-retweeted total number of tweets. The collection of tweets was facilitated through the Twitter API using Python's Tweepy library. A query with the keyword “demonstratie” (demonstration in Dutch) was executed. Further filters were applied to exclude retweets (via the statement -filter:retweets) and only select Dutch tweets (by setting the lang=‘nl’ parameter within the Tweepy.cursor function). To use the Twitter API, users must provide a ‘consumer key’ and ‘consumer secret’, which are obtained through the Twitter developers’ page (https://developer.twitter.com/en/docs/authentication/oauth-1-0a/api-key-and-secret).

**Table 3:** Number of tweets collected per day (based on the tweet *Created_At* timestamp (which is made time zone aware).

*Date*	Counts
2020-05-31	1,531
2020-06-01	27,174
2020-06-02	28,648
2020-06-03	12,096
2020-06-04	6,468
2020-06-05	3,697
2020-06-06	3,257
2020-06-07	2,030

### 4.2 Data pre-processing

The data analysis focused exclusively on the textual content of tweets, disregarding meta information such as geolocation, likes, retweets, and comments. Textual constructs such as hashtags, emojis, and punctuation were retained in the data sets. Additionally, textual representations of personal information (e.g., mentions) underwent hashing, ensuring that equivalent hashtags were replaced by the same hash. Subsequent refinements in data processing included the implementation of more specific filters to narrow down the dataset to tweets explicitly associated with “Black Lives Matter” and “Amsterdam”. In the labelling phase, tweets deemed off-topic, containing personal information (e.g., names, addresses, phone numbers), duplicates, and those identified as originating from known bots were systematically excluded from the data.

### 4.3 Labelling process

The process of labelling tweets continued for 18 months (within the years 2022 and 2023), this data was the first of four sets. Since the label team started with this set, measures to ensure high-quality labels were implemented. Initially, a labelling protocol was drafted to include detailed descriptions of the demonstrations and a formal definition of each label including constraints and example tweets. After the labellers received their protocol, a brief introductory training session was held to familiarize the labellers with the tooling and tasks, addressing any questions about the protocol and tools. After this training session, a pilot study was conducted to see if all labellers understood the task and could work with the tooling. For this pilot, a set of pre-labelled tweets was assigned to the labellers to check if the labels correspond to the expectations.

During the labelling period that followed, the team could discuss difficult-to-label tweets and create consensus on how to handle certain edge cases. The experimenter was in contact with OSINT to discuss preliminary results and ask the labellers’ questions. During the weekly label meetings, statistics were shared with the labellers on the ratio of used labels per labeller and the average speed of the labelling, to get a more evenly distributed work process. Note that the labelling statistics are included in the data, with hashed usernames. The labelling speed records served an additional purpose later in the labelling process since any optimizations on the labelling tool could be monitored in terms of increased labelling speed. Finally, the labelling protocol was also updated when specific rules or definitions were added or updated based on ongoing experience with the labelling tasks. The final version of the label protocol is included as a supplement to this paper.

During label meetings, additional data-related discussions occurred, such as the proposal to maintain lists of referenced media sources categorized by type (mainstream or not and verified or not). Although maintaining these lists costs time, the lists helped labellers in the long run to maintain a consistent label process, for example when a labeller encountered a news source and was not sure whether to label the source as mainstream media or not, he/she only had to look at the list to determine which specific label was associated with the source. Discussions also covered criteria for excluding or discussing tweets, as some discuss-tweets were easily labelled with additional rules.

When all tweets were labelled, the experimenter checked the labelled tweets manually to look for obvious mistakes, i.e. if a tweet in a non-Dutch language was excluded, after which the post-processing started. This resulted in the assigned labels as presented in Table 4, where the number of labelled tweets per labeller is presented in Table 5. Note that some labels are more common than others, which might indicate that some of the labels cover a more specific area than others within this set. Also note that the number of tweets differs between labellers which are presented in Table 5.

**Table 4:** Labels of the 2022 data set including the number of occurrences.

Label	Count
has_Not_Event_Related	5,620
has_Not_Incident_Related	5,341
has_Low_Priority	4,769
has_Non_Relevant	4,032
has_Relevant	2,102
has_Incident_Related	500
has_Corona	494
has_High_Priority	335
has_Event_Related	185
has_Riot	1
has_Violence	1

**Table 5:** Number of tweets that are labelled per labeller. Note that the discuss and exclude tweets are not included. These counts do not include pre-labelled tweets that have not been updated by the user.

*labeller*	Tweet Count
9f43	3,703
83de	1,427
f3e7	516
5034	388
1dd8	99
5d9d	22

### 4.4 Labelling tool: Tweeti

To label the tweets, a specially designed tool named Tweeti (Source code of the label tool Tweeti can be found at https://github.com/LMuter/Tweeti), an abbreviation for ‘Twitter Intelligence’, is developed (see Fig. 2). With this tool, a labeller can view the text of a randomly selected tweet and assign the appropriate label. Selecting a label is done by using the button on the bottom line of the Tweeti tool. The label buttons can be single (one click resolves a single label) or can be hierarchical (after clicking a menu will appear to select one or more labels within a category). When a label is selected it is displayed above the label buttons in a colour that depends on the label's category (these colours can be changed in the admin view). After a label is assigned, the labeller can also withdraw the label by clicking on the x-symbol in the corner of the assigned label. Before a label button is pressed, a labeller can select a part of the tweet text that will be annotated when assigning a label. These annotations can be viewed by hovering over the label.

The bottom row contains the function buttons, which include the Previous tweet and Next tweet buttons to either move to the next or previous tweet to label, the Discuss button to select the current tweet for the weekly discussion session, and the Exclude button to indicate that the current tweet is not relevant and should be excluded from the set, note that labellers cannot delete tweets, only users with admin privileges can do that.

The next tweet button is used to randomly select a new tweet. When the labeller does not assign any label, the No Label is automatically assigned. With the previous button, a labeller can go back into the labelling history of the current session. However, after the browser is closed, this history will be erased.

For all end-user functionalities, there are shortcuts available to speed up the labelling process (more experienced labellers can build up muscle memory which can make the process of labelling even more efficient). Note that Tweeti keeps track of all label changes, for example when a user selects label A and then removes label A and selects label B, this is recorded as three separate transactions.

The labeller view also contains a menu where the labeller can see the shortcut keys and some basic statistics such as the time it took to label the current tweet, and the number of tweets labelled during the current session.

Finally, Tweeti allows users to log in and out, and update or reset their password. Specific user information, for example, email and password can be changed within the admin view. On the admin side, there are functionalities to update tweets and their associated labels, add and remove labellers, and download and upload CSV files that represent tweets. When uploading tweets, the admin user can also add a category that can be referred to when labelling multiple sets. The admin view also allows uploading pre-labelled tweets and changing the label's colour and order in which the labels are presented.

The Tweeti tool is dockerized and available on GitHub, there is an installation instruction available, and the repository includes user and admin manuals.

### 4.5 Experimental design

After the tweets are collected, the full tweet texts are stored in the Tweeti labelling tool for further processing.

In Tweeti, a randomly selected tweet is presented to a labeller, who can associate labels with the selected tweet. The labels are divided into different categories (Relevance, Media, Event Related, Incident Related, and Behavioural) other labels include Related Topic and No Label. A labeller can select a single or multiple labels per category, depending on the settings per category. This feature is used to allow the labeller to add the generic- or related topic label to indicate that a given tweet contains contextual information about the demonstration, but not about the demonstration itself (for example when a tweet refers to another Black Lives Matter demonstration in a different city). The user is allowed to use zero or more labels from the Relevance-, Media-, Event Related-, IncidentRelated-, and Behavioural categories, but every labelled tweet is permitted to have at least a single label. Thus, when a labeller presses the next key when no label is provided, the no-label is automatically applied to the selected tweet. Some labels cannot be chosen for the same tweet, for example when a labeller tries to add No Label when Related Topic is already set, Tweeti ignores the action.

Tweets referring to a non-related topic or written in a language other than Dutch could be excluded by the labeller. A labeller is allowed to use the back button, so it is possible to change previously labelled tweets based on new insights from other tweets. After a session is closed, the labelled tweets receive a final status, so they are included in the final data set and not shown in other sessions, or depending on the settings, remain in their active status to allow the tweets to be displayed in a different session. tweets that are very difficult to label can be added to a discussion list, where tweets on this list are discussed during the weekly meeting with the labelling team. After a tweet is discussed, the label is manually updated by using Tweeti's update function and finalized so it is included in the final data set.

In addition to the label, a labeller can also annotate the text which is associated with a given label. Although we did not include the annotated text, we did use the annotations when samples of labelled tweets were checked.

## Limitations

The labelling of this data consisted of two separate teams at different moments in time. In 2022, a team of five labellers classified the tweets using multiple labels. In 2023, a team of three members classified the tweets using a single binary label, since the expression of discomfort label could be useful to predict sentiment and was not included during the 2022 labelling. Apart from the experimenter, only a single labeller joined both the 2022 and 2023 team.

**Fig. 1:**
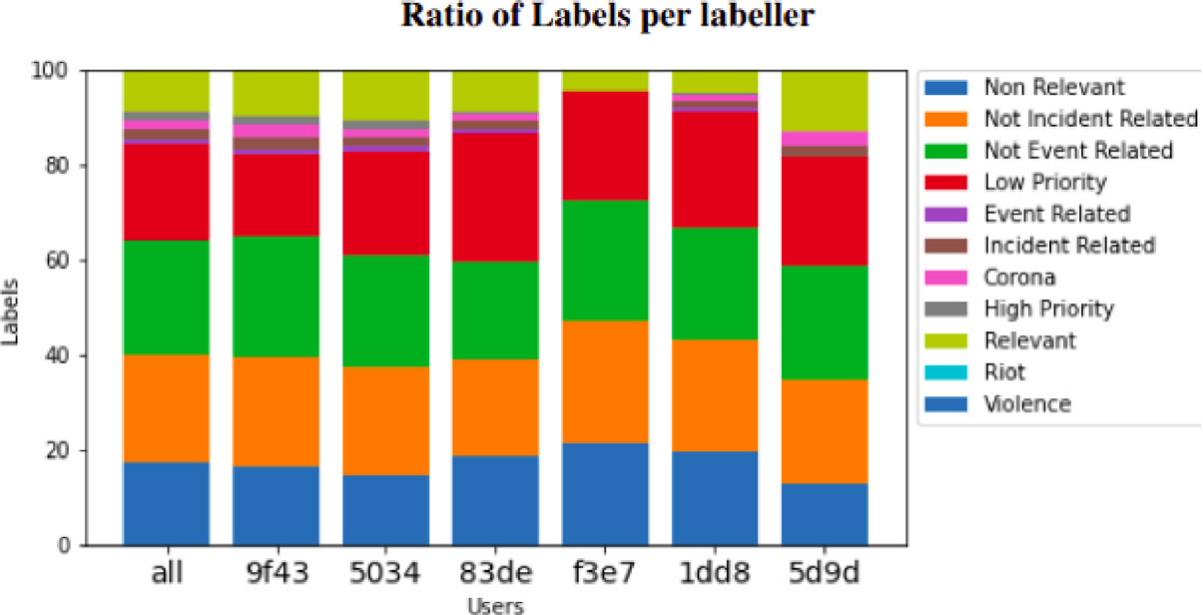
This figure represents the ratio of label counts per labeller. The labeller names are hashed. The leftmost column represents the ratio of all labels. Note that the number of labelled tweets differs between labellers, see Table 5.

Another limitation is that only a subset of the tweets is labelled by more than one labeller which resulted in an average inter-labeller reliability of 0.70. Thus, founded inter-labeller reliability might diverge from inter-labeller reliability if the entire set. The reason that only a subset was labelled by more than one labeller is that when the experimenter included the same tweet for multiple labellers, questions were raised by labellers about duplicate tweets in the label meeting that followed. This indicated that labellers on the one hand discussed tweets alongside the label meetings and on the other hand, made each other aware of tweets that are difficult to label. To tackle the problem of low inter-labeller reliability even more, the experimenter presented a graph that showed the ratio of labels for each labeller during each weekly label meeting (see Fig. 1). In this ratio graph, the ratio of labels used by labellers moved toward the averages when the labelling progressed.

**Fig. 2:**
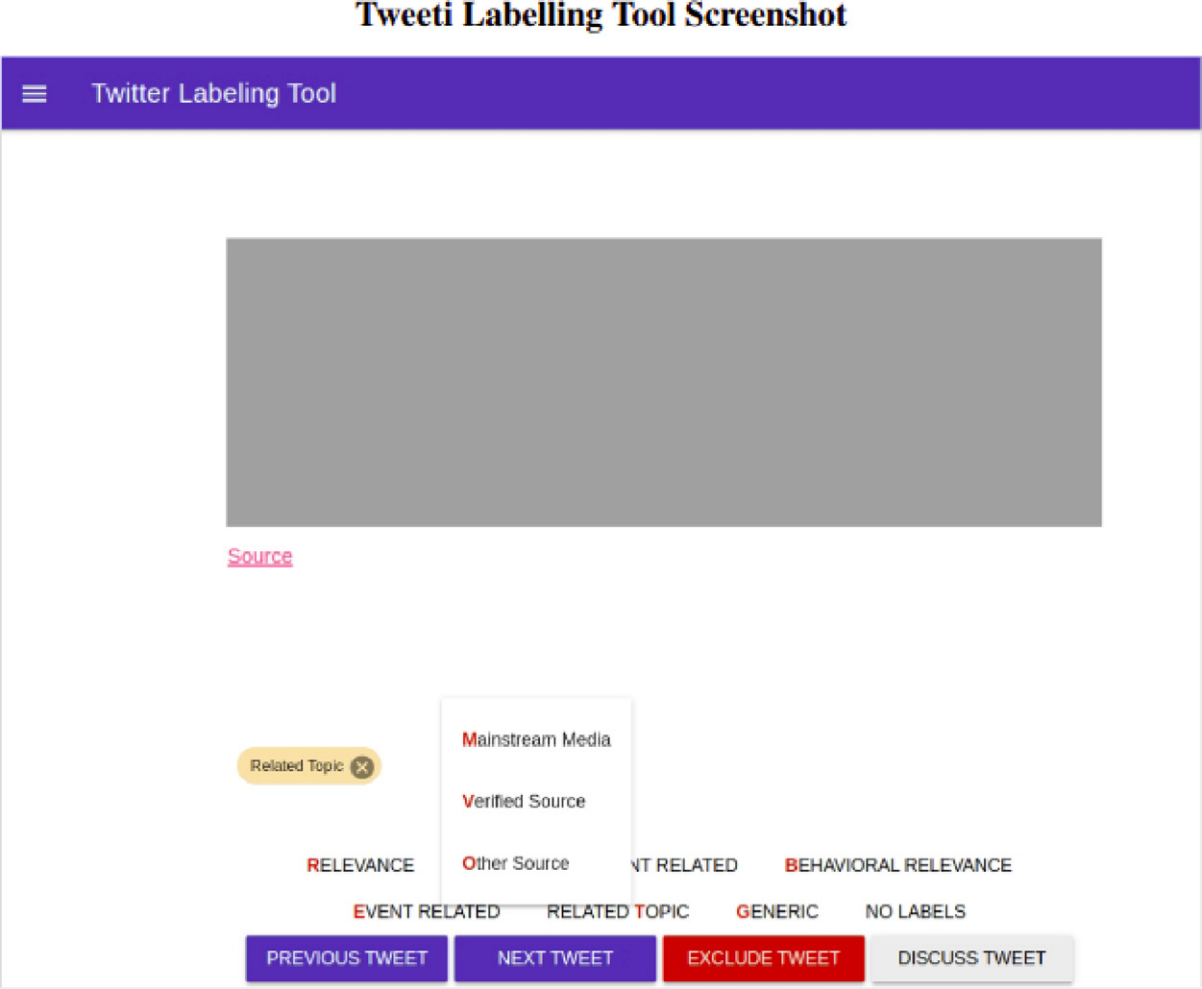
Representation of the Tweeti labelling tool used to label the majority of the tweets. The figure shows the label categories (Relevance, Incident Related, and Behavioural Relevance), the labels (Mainstream Media, Verified Source, and Other Source), a selected label (Related Topic), and the function buttons (Previous Tweet, Next Tweet, Exclude Tweet, Discuss Tweet). Note that the content of the displayed tweet is covered by a grey square.

Finally, the size of the data is limited since only a fraction of the entire set is labelled. There might be bias within the labels since every labeller has a Dutch background. The balancedness of the labels caused a challenge in training the BERT-models which could have impacted the results.

## Ethics Statement

Using data from social media platforms (e.g. Twitter) poses ethical concerns regarding consent. Since the tweets are originally posted by Dutch citizens, any usage of this data automatically falls under specific rules (such as the GDPR). The GDPR includes strict rules that individual users cannot be traced from this data [8] and any action of gathering personal information should have a clear purpose, restricted to additional regulations on conditions such as storage, usage, and privacy. For this research, information regarding storage and usage is included within a data management plan. In line with these rules, we want to point out to authors who want to use any of the provided data sets that they need to determine whether the data collection needs ethical approval from appropriate institutional review boards and ethics committees (for more information, contact https://www.uu.nl/en/organisation/practical-matters/privacy/data-protection-officer). Particular attention is needed to determine if informed consent from possible identifiable participants is needed or to ensure that these are properly anonymized, for example, hashtags could point to a specific public spokesman or have the potential to lead to certain groups. Secondly, authors need to consider the data redistribution policies from the platform used to gather information and ensure they have the right to share these data. Policies vary among platforms and are updated regularly, therefore it is the authors’ responsibility to ensure that their work complies with the platforms’ policies.

## CRediT authorship contribution statement

**Laurens H.F. Müter:** Conceptualization, Methodology, Software, Validation, Formal analysis, Investigation, Resources, Data curation, Writing – original draft. **Christof van Nimwegen:** Supervision. **Remco C. Veltkamp:** Supervision.

## Data Availability

Dutch Tweets Before, During, and After a Black Lives Matter Demonstration in Amsterdam: Expert Annotated Data, Protocol, and Labelling Tool (Original data) (yoda)
